# Steps to recovery: body weight-supported treadmill training for critically ill patients: a randomized controlled trial

**DOI:** 10.1186/s13063-020-04333-y

**Published:** 2020-05-15

**Authors:** Robin C. H. Kwakman, Juultje Sommers, Janneke Horn, Frans Nollet, Raoul H. H. Engelbert, Marike van der Schaaf

**Affiliations:** 1 Department of Rehabilitation, Amsterdam Movement Sciences, Amsterdam UMC, University of Amsterdam, Meibergdreef 9, Amsterdam, Netherlands; 2 Faculty of Health, Center of Expertise Urban Vitality, Amsterdam University of Applied Sciences, Amsterdam, The Netherlands; 3 Education of Physiotherapy, University of Applied Sciences Amsterdam, Amsterdam, The Netherlands; 4 Department of Intensive Care, Neurosciences, Amsterdam UMC, University of Amsterdam, Meibergdreef 9, Amsterdam, Netherlands

**Keywords:** ICU, intensive care, rehabilitation, physical function, body weight-supported treadmill, critically ill, ambulation, intervention

## Abstract

**Background:**

Early mobilization has been proven effective for patients in intensive care units (ICUs) to improve functional recovery. However, early mobilization of critically ill, often mechanically ventilated, patients is cumbersome because of the attachment to tubes, drains, monitoring devices and muscle weakness. A mobile treadmill with bodyweight support may help to initiate mobilization earlier and more effectively. The aim of this study is to assess the effectiveness of weight-supported treadmill training in critically ill patients during and after ICU stay on time to independent functional ambulation*.*

**Methods:**

In this randomized controlled trial, a custom-built bedside body weight-supported treadmill will be used and evaluated. Patients are included if they have been mechanically ventilated for at least 48 hours, are able to follow instructions, have quadriceps muscle strength of Medical Research Council sum-score 2 (MRC 2) or higher, can sit unsupported and meet the safety criteria for physical exercise. Exclusion criteria are language barriers, no prior walking ability, contraindications for physiotherapy or a neurological condition as reason for ICU admission. We aim to include 88 patients and randomize them into either the intervention or the control group. The intervention group will receive usual care plus bodyweight-supported treadmill training (BWSTT) daily. The BWSSTT consists of walking on a mobile treadmill while supported by a harness. The control group will receive usual care physiotherapy treatment daily consisting of progressive activities such as bed-cycling and active functional training exercises. In both groups, we will aim for a total of 40 minutes of physiotherapy treatment time every day in one or two sessions, as tolerated by the patient. The primary outcome is time to functional ambulation as measured in days, secondary outcomes include walking distance, muscle strength, status of functional mobility and symptoms of post-traumatic stress. All measurements will be done by assessors who are blinded to the intervention on the regular wards until hospital discharge.

**Discussion:**

This will be the first study comparing the effects of BWSTT and conventional physiotherapy for critically ill patients during and after ICU stay. The results of this study contribute to a better understanding of the effectiveness of early physiotherapy interventions for critically ill patients.

**Trial registration:**

Dutch Trial Register (NTR) ID: NL6766. Registered at 1 December 2017.

## Introduction and rationale

There is an increasing amount of evidence stating that early mobilization and activation of patients admitted to the intensive care unit (ICU) improves functional recovery [1–7]. Current practice may include active sitting and standing up exercises, cycling on a cycle ergometer with upper or lower limbs (while in bed or in a chair) and ambulation training whenever possible. However, practicing walking with critically ill patients after a longer period of mechanical ventilation on the ICU is demanding because of muscle weakness and the attachment of patients to mechanical ventilation, drains, infusion lines and monitoring equipment [8]. Moreover, ambulating critically ill patients puts a large demand on staff as it often requires at least three persons and a considerable amount of time [9]. Innovation and new technological developments may facilitate ambulation training in patients experiencing these difficulties and may also improve the feasibility, safety and effectiveness of early mobilization in the intensive care unit [10, 11].

Body weight-supported treadmill training (BWSTT) has shown to be an effective modality for improving fitness, walking capacity and daily functioning in different rehabilitation populations with muscle weakness [12–14]. In a previous pilot study, we showed that BWSTT was feasible and safe in critically ill patients [12]. Early ambulation training on the ICU supported by BWSTT can potentially shorten the time to independent ambulation for critically ill patients and improve their functional status at ICU and hospital discharge.

The aim of this study is to assess the effect of BWSTT in critically ill patients during ICU and hospital stay on time to independent functional ambulation as compared to usual care.

We hypothesize that BWSTT leads to earlier independent functional ambulation compared to usual care.

## Methods

### Study design

A monocenter randomized controlled trial (RCT) will be executed on the ICU and general wards of the Amsterdam University Medical Center, location AMC (Amsterdam UMC) between May 2018 and September 2020. The Amsterdam UMC houses a mixed medical and (neuro) surgical ICU with 26 beds.

### Eligibility criteria

As we aim to include patients who are at risk for prolonged rehabilitation, we focus on patients with a longer duration of mechanical ventilation [15–17].

#### Inclusion criteria

Participants have to be mechanical ventilated for ≥ 48 hours, must be responsive and able to follow instructions according to the Short 5-item Questionnaire (S5Q 5 out of 5) [18], have quadriceps muscle strength ≥ 2 according to the Medical Research Council [19] and must be able to sit unsupported on the edge of the bed.

#### Exclusion criteria

Exclusion criteria are; the presence of contraindications for physiotherapy according to the evidence statement for ICU physiotherapy [18] such as heart rate of < 40 or > 130 beats per minute, a recent myocardial ischemia or a mean arterial pressure (MAP) of < 60 or > 110 mmHg, surgical contraindications (i.e. instable fractures, craniotomy, open abdomen or thorax), insufficient knowledge of the Dutch language, inability to walk independently in the month prior to ICU admission, one or more amputated lower extremities, mental retardation and when death is imminent. Patients with neurological diseases and disorders as indication for ICU admission are excluded because underlying pathology may affect recovery of functional walking ability.

#### Recruitment

All patients admitted to the ICU for > 48 hours will be screened daily for eligibility.

Patients who fulfil the inclusion criteria will be invited to participate in the study. In case of severe muscle weakness and inability to provide written informed consent, verbal consent will be obtained, and an independent witness will sign on behalf of the participant. Participants can leave the study at any time for any reason if they wish to do so without any consequences.

### Randomization and blinding

We will use a variable block randomization method performed by the software CASTOR EDC, a Good Clinical Practice (GCP)-approved online software service. These variable blocks are of various sizes. This way the researcher does not know how large the blocks are at any given moment, and thus cannot predict the next randomization. After randomization, the patient identification number (PIN) number and treatment allocation will be provided to the physiotherapist performing the interventions. Due to the kind of the intervention, it is not possible to blind physiotherapists and participants for the intervention. The primary outcome will be assessed by a researcher who is blinded for the intervention. Secondary outcomes will be assessed by blinded researchers except during the stay of the participants in the ICU where measurements will be assessed by the physiotherapist during treatment (usual care and intervention) to avoid too much strain on the patients.

### Treatment of subjects

#### Treatment before enrollment

Patients will be screened for eligibility at a daily basis. Patients who do not meet the criteria to enter the study but are considered potentially eligible for inclusion at a later time, will receive physiotherapy according to usual care but no BWSTT. Potentially eligible patients are those patients who are unable to perform ambulation exercises out of bed at the time of screening but are likely to improve over time. They may be sedated, hemodynamically instable, have delirium and/or too severe muscle weakness to be mobilized. For these patients, usual care physiotherapy consists of passive and (guided) active exercises in bed, passive and partially active cycling in bed if possible, inspiratory muscle training (IMT), transfer training to the bedside and active balance training in a seated position [7, 18, 20]. Training load in terms of frequency, intensity, type and time will be adjusted according to the physical capacity of the patient.

#### BWSTT intervention

**Fig. 1:**
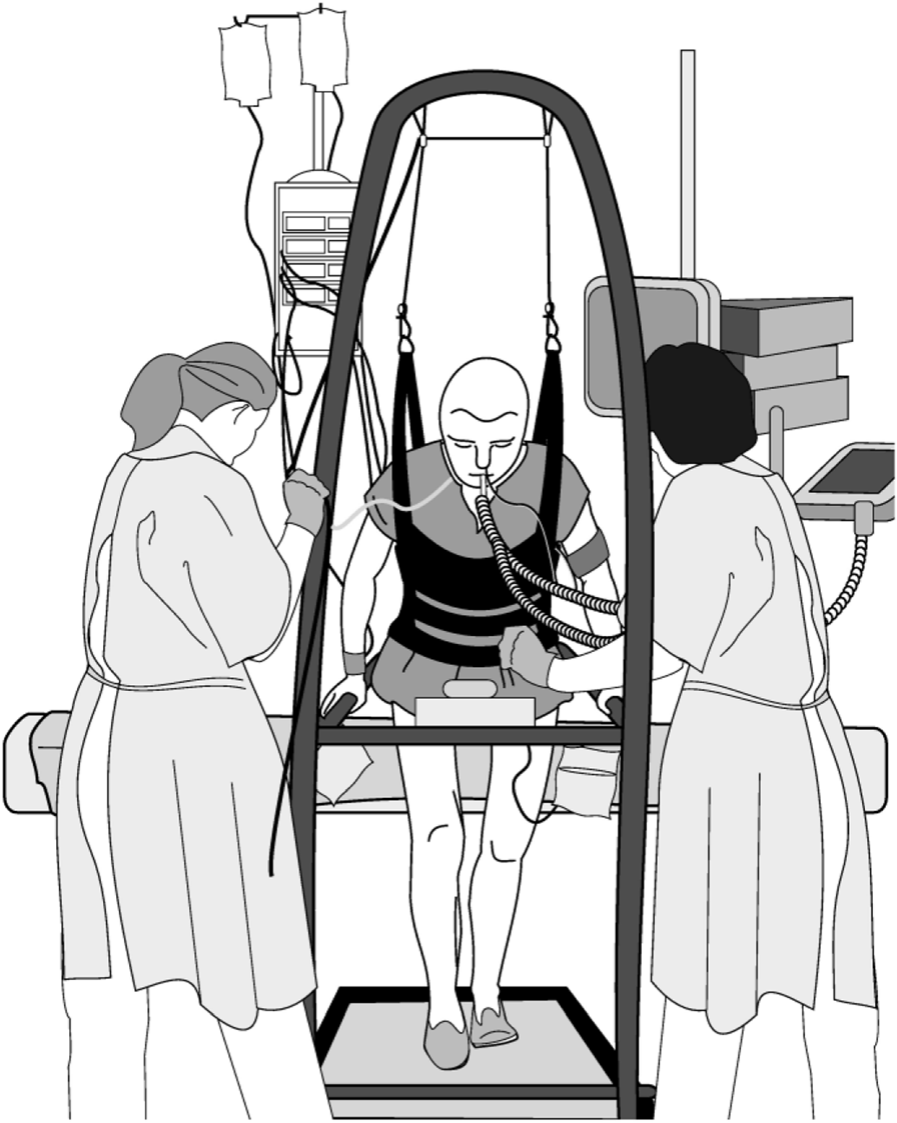
Schematic illustration of the BWST

Participants in the intervention group will receive BWSTT in addition to usual care. The BWSST intervention consists of walking on a treadmill while supported by a harness (see Fig. 1 for a schematic illustration of the BWST).

A mobile treadmill with weight-bearing utility will be used for BWSTT (Fig. 1). The body weight-supported treadmill enables early ambulation in patients with insufficient motor control or muscle strength to fully bear weight. The treadmill has been developed in the Amsterdam UMC.

The intervention will be provided by trained physiotherapists. BWSTT is provided on a daily basis for 5 days per week (not during the weekend) in the ICU or medium-ICU (MICU) and the regular ward.

For each session, two physiotherapists are involved. Before the BWSTT, the patient is seated upright at the side of the bed and the harness is fitted. The treadmill is positioned at the bedside and the harness is fastened to the safety lines attached to the frame of the BWST to support bodyweight. One physiotherapist stands next to the BWST to adjust the amount of bodyweight support and to monitor vital functions at the bedside monitor. The second physiotherapist guides the patient and coordinates the intervention in terms of walking speed and distance depending on the walking capacity of the patient. The aim is to have the patient walk at a self-chosen comfortable walking speed. The intervention stops as the vital signs exceed the safety criteria [18], the patient indicates that he has reached his maximum load, or according to the clinical observation of the patient by the physiotherapist. After a period of recovery, if vital signs and perceived exertion return to pre-exercise levels, the physiotherapist may decide to continue BWSTT in consultation with patient and medical staff in order to reach a sufficient training load. This exercise can be repeated multiple times before the intervention is ended. When the intervention is ended the harness is removed while the patient is seated at the bedside, the patient will then be guided back to a supine position in bed.

After ICU discharge, BWSTT will be continued daily on the regular ward until the patient is able to ambulate with walking aid and minimal physical support for balance assistance (Functional Ambulation Categories [FAC] ≥ 2). Physiotherapy according to usual care will be provided from that point on until hospital discharge.

#### Usual care

Concerning impairments in body functions and structures usual care physiotherapy goals are to prevent and treat impairments in range of motion, muscle tone, muscle mass, generalized and respiratory muscle strength, the cardiorespiratory system, and exercise capacity. Concerning impairments in activities, physiotherapy goals are to improve functional status, balance, transfers, and walking capacity. Active physiotherapy consists of progressive active exercises in bed or on a chair, active ergometer cycling, walking, active range of motion exercises, transfer training, balance training, active functional training exercises, and IMT [18].

The treatment plan for critically ill patients with at least Medical Research Council sum-score 2 (MRC 2) in the lower extremities will focus largely on progressive (guided) active and functional training. Passive physiotherapy is seldom applied since active physiotherapy interventions are preferred in responsive patients. Starting from MRC 3 in the lower extremities, treatment will also focus on standing up and walking.

Various exercise equipment is applied during physiotherapy treatment. Weights, elastic resistance bands, and computer games will be used to assist in active exercises. We will use walking frames, walkers, active lifts, and other aids to assist in training balance, transfers, and walking.

Usual care consists of one or multiple individual supervised physiotherapy sessions on a daily basis 7 days per week. The physiotherapist decides which exercises are administered based on the current (medical) status, the preference and tolerance of the patient at the time of the treatment session and available guidelines regarding evaluation of safety criteria and clinical observations [18]. These physiotherapy sessions will be performed by experienced ICU physiotherapists and will be continued on the regular wards after ICU discharge until hospital discharge or until pre-admission functional status is achieved and all treatment goals have been reached.

#### Main study endpoint

The main study endpoint is the number of days to functional ambulation of 3 or higher as measured with the FAC. The FAC categorizes patients according to basic motor skills necessary for functional ambulation [21]. FAC categories are rated as follows:

(0) The patient cannot walk, or needs help from two or more persons, (1) the patient needs firm continuous support from one person who helps carrying weight and with balance, (2) the patient needs continuous or intermittent support of one person to help with balance and coordination, (3) the patient requires verbal supervision or stand-by help from one person without physical contact, (4) the patient can walk independently on level ground, but requires help on stairs, slopes, or uneven surfaces, (5) the patient can walk independently anywhere.

The FAC will be assessed daily until hospital discharge by a researcher blinded to the intervention. Should a patient still receive BWSTT after transfer to the regular ward, the study intervention, and the blinded outcome assessment will be performed separately. If a patient is discharged to another hospital before the main study endpoint (FAC 3) is reached, the assessment of walking capacity will be continued until a FAC score of 3 has been reached.

#### Secondary endpoints

**Fig. 2:**
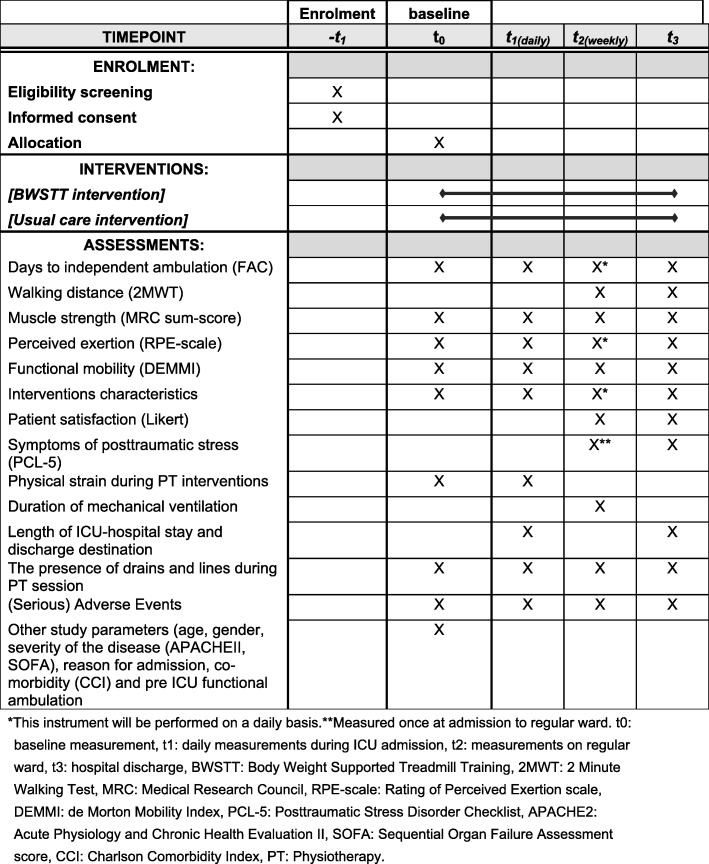
Schedule of enrollment, interventions, and assessments (SPIRIT)

The secondary endpoints are walking distance as measured with the 2 Minutes Walking Test (2MWT) [22], muscle strength of upper and lower extremities as measured with the Medical Research Council sum-score (MRC sum-score) [19], perceived exertion after physiotherapy interventions as measured with the Rating of Perceived Exertion scale (RPE scale) [23], physical strain during physiotherapy interventions as measured by peak values of heart rate, mean arterial pressure, respiratory frequency and oxygen saturation, functional mobility as measured with the De Morton Mobility Index (DEMMI) [24, 25], duration of mechanical ventilation, ICU, and hospital length of stay, discharge destination, symptoms of post-traumatic stress as measured with the Posttraumatic Stress Disorder Checklist (PCL-5) [26] and patient satisfaction as measured with a 5-point Likert scale. (Serious) Adverse Events (S) AEs related to the study intervention will be recorded in accordance with good clinical practice guidelines and legislation. Physiotherapy intervention characteristics including numbers of staff and time needed, frequency and content of treatment and walking time, distance and support needed during BWSTT will be recorded for both groups. Outcomes will be assessed at different points in time as is shown in Fig. 2.

Other patient characteristic including age, gender, severity of the disease [27, 28], reason for admission, co-morbidity [29], pre-ICU functional ambulation, and the presence of drains and infusion lines will be recorded from the patient files.

#### Data storage and archiving

A Trial Master File containing all study documents will be compiled in accordance with GCP guidelines and the Dutch Medical Research Involving Human Subjects Act (WMO) and will be stored safely [30]. These documents will only be accessible by research staff directly involved in this study. Castor EDC (Amsterdam, The Netherlands) will be used as online database, we will use digital case report forms to collect and store data.

After the end of the study all documents will be archived for a period of 15 years.

All included patients are identified by a PIN. A list of PIN/name combinations will be securely stored by the head investigator.

A privacy impact analysis has been performed and approved by the Amsterdam UMC’s privacy officer in order to warrant confidentiality of privacy before, during, and after the trial.

#### Monitoring and Data Safety Monitoring Board

In this study a non-CE certified medical device (mobile treadmill) will be used. This study is classified as a moderate risk study by the Amsterdam UMC (AMC) local Ethics Committee and will be monitored by a certified monitor according to Amsterdam UMC monitoring policy. A Data Safety Monitoring Board (DSMB) has been installed consisting of an independent intensivist, epidemiologist, and a statistician. Specific tasks of the DSMB are to monitor safety data to guide recommendations for continuation of the study or early termination because of clear harm, to evaluate the overall conduct of the trial and to monitor sample size assumptions.

#### Adverse events

(S) AEs potentially related to the study intervention, reported to, or observed by the study personnel will be documented in the source documents. For SAEs, this includes any untoward medical occurrence, event or effect related to the study intervention that at any dose is life-threatening, results in death, prolonged hospitalization, significant disability or incapacity and the occurrence of falls, wounds, dislocation of mechanical ventilation, and arterial lines. For each, potentially study-related (S) AE, the time of onset and the time when resolved, type, any action taken and intensity will be recorded in the case record form (CRF). Evaluation of outcome, seriousness, period, treatment, and the relationship with the study (causality) will be classified for each study related (S) AE. All study related (S) AEs of subjects being part of the analysis will be listed and described in the report.

#### Ethics and dissemination

The study will be conducted according to the principles of the Declaration of Helsinki following GCP rules.

As stated in the current version of Fortaleza, Brazil, 2013 and in accordance with the Dutch Medical Research Involving Human Subjects Act (WMO).

The study protocol has been approved by the Amsterdam UMC (AMC) local Ethics Committee (NL63104.018.17 - METC 2017_225). The study has been registered at the Dutch Trial Register, ID: NTR6943.

#### Statistical analysis

Sample size was calculated using data from previous studies where BWSTT [12] and usual care [24] was provided to critically ill patients in the Amsterdam UMC. Data of 35 patients were retrieved. On average, these patients reached the endpoint (≥ FAC 3) at day 15 after the inclusion criteria were met. In the sample data, we found a hazard ratio of 1.87 at 15 days in favor of BWSTT. All patients reached the endpoint within the study period. Given a type 1 error rate of 5% and a power of 0.8 the analysis resulted in a required sample size of 88 (44 + 44) patients before correction for attrition, assuming a 10% dropout rate. A check on sample size assumptions will be performed after inclusion of 40 participants as the dataset used for sample size to calculate was relatively small.

The primary objective of this study is to assess the effect of BWSTT in critically ill patients on time to functional ambulation as compared to usual care. For this, Kaplan-Meier analyses will be performed and comparisons between both groups will be made. For the primary analysis we will use intention to treat principles. Multiple imputation will be applied in cases where the primary outcome is missing. Cox regression analysis will be performed when imbalances between groups are found at baseline in age (mean difference ≥ 10%), muscle strength (MRC sum-score mean difference ≥ 10%), functional ambulation level (FAC mean difference > 1) and co-morbidities (Charlson Comorbidity Index [CCI] mean difference > 1) as they are potentially related to the primary outcome. Significance values are not considered when determining baseline differences.

The secondary objective of this study is to analyze between group differences of other patient relevant outcome measures for the evaluation of BWSTT. Patient characteristics and secondary study endpoints will be described using descriptive statistics according to their distribution. If the variables are normally distributed, they are expressed as mean and standard deviation (SD). Skewed variables are presented with medians and interquartile ranges (IQR). Between groups comparisons will be made using the appropriate tests according the variable type and distribution.

For this study, analysis will be performed using the software package R version 3.6.1.

## Discussion

BWSTT has shown to be an effective modality for ambulatory function in different rehabilitation populations with muscle weakness [12–14]. This might indicate that BWSTT could also benefit critically ill patients in their recovery to independent function ambulation. However, it has never been evaluated in critically ill patients. Early ambulation training in critically ill patients on the ICU supported by BWSTT can possibly facilitate and shorten the first time to ambulation and improve functional status at ICU and hospital discharge [12]. Another potential beneficial factor of BWSTT is that less time and staff are needed for ambulation training as a patient can stay at the bedside. Furthermore, BWSTT can provide a mental boost as making the first steps after or during critical illness can provide a patient with a more positive future perspective and more self-confidence, especially for those patients who have been at the ICU for an extended amount of time. Early ambulation training using BWSTT within a group of fragile critically ill patients should be evaluated thoroughly on safety and efficacy before implementing in usual care.

A possible limitation of the Steps to Recovery trial is blinding of group assignment for outcome assessors on the ICU. Assessments on the ICU cannot be blinded because having a blinded assessor assess all outcomes on the ICU would be too demanding for patients in addition to the interventions given. Despite the challenges in blinding outcome assessors on the ICU, it is important to note that it is expected that the primary outcome (time to independent ambulation) will be reached on the regular wards where blinded assessments are performed. Another possible limitation is the generalizability of these study results as we have only included a limited part of the ICU population and excluded neurological patients, albeit for a good reason as this underlying pathology may affect recovery of functional walking ability.

In summary, the Steps to Recovery trial is the first randomized controlled trial to evaluate the effects of BWSTT on recovery time to functional ambulation in critically ill patients. The results of this study will add to the knowledge of early rehabilitation and recovery of critically ill patients.

### Trial status

Based on protocol version 3, February 12, 2018. This trial has begun to enroll patients from May 2018. Recruitment will be continued until September 2020.

## Data Availability

The datasets generated and/or analyzed during the current study will be available from the corresponding author on reasonable request.

## Abbreviations

AE: Adverse event
APACHE II: Acute Physiology and Chronic Health Evaluation II
BWSTT: Body weight-supported treadmill training
CRF: Case record form
DEMMI: de Morton Mobility Index
DSMB: Data Safety Monitoring Board
FAC: Functional Ambulation Categories
GCP: Good Clinical Practice
ICU: Intensive care unit
IMT: Inspiratory muscle training
METC: Medical Research Ethics Committee (MREC) / in Dutch: medisch ethische toetsing commissie
MICU: Medium intensive care unit
MRC: Medical Research Council
PT: Physiotherapy
PIN: Patient identification number
RCT: Randomized controlled trial
(S)AE: (Serious) adverse event
S5Q: Short 5-term Questionnaire
WMO: Medical Research Involving Human Subjects Act
